# The impact of colonialism on head and neck cancer in Brazil: a historical essay focussing on tobacco, alcohol and slavery

**DOI:** 10.1016/j.lana.2024.100690

**Published:** 2024-02-09

**Authors:** Beatriz Nascimento Figueiredo Lebre Martins, Erison Santana Dos Santos, Felipe Paiva Fonseca, William Nassib William, Thiago Bueno de Oliveira, Gustavo Nader Marta, Aline Lauda Freitas Chaves, Ana Carolina Prado-Ribeiro, Olalekan Ayo-Yusuf, Maria Paula Curado, Alexandre Macchione Saes, Luiz Paulo Kowalski, Alan Roger Santos-Silva, Latin American Cooperative Oncology Group, Brazilian Group of Head and Neck Cancer, William Nassib William, Thiago Bueno de Oliveira, Gustavo Nader Marta, Aline Lauda Freitas Chaves, Maria Paula Curado, Luiz Paulo Kowalski, Alan Roger Santos-Silva

**Affiliations:** a University of Campinas (UNICAMP), Oral Diagnosis Department, Piracicaba Dental School, Piracicaba, São Paulo, Brazil; b Federal University of Minas Gerais, Department of Dental Clinic, Pathology and Surgery, Belo Horizonte, Brazil; c Grupo Oncoclínicas, São Paulo, Brazil; d Latin American Cooperative Oncology Group, Brazilian Group of Head and Neck Cancer, Brazil; e Medical Oncology Department, AC Camargo Cancer Center, São Paulo, SP, Brazil; f Department of Radiation Oncology, Hospital Sírio-Libanês São Paulo, São Paulo, Brazil; g DOM Oncology Group, Divinópolis, Minas Gerais, Brazil; h São Paulo State Cancer Institute (ICESP-FMUSP), Dental Oncology Service, Brazil; i Serviço de Medicina Oral, Hospital Sírio-Libanês, São Paulo, Brazil; j Africa Centre for Tobacco Industry Monitoring and Policy Research, School of Health Systems and Public Health, University of Pretoria, Pretoria, South Africa; k Epidemiology AC Camargo Cancer Center, São Paulo, SP, Brazil; l Professor of Economic History, Department of Economics, University of São Paulo, Brazil; m Department of Head and Neck Surgery and Otolaryngology, A C Camargo Cancer Center, São Paulo, Brazil; n Department of Head and Neck Surgery, University of São Paulo, Brazil

**Keywords:** Cancer, Head and neck neoplasms, Epidemiology, Brazil, Ethnicity, Prognosis, Mortality, Tobacco, Alcohol, Colonialism

## Abstract

Colonialism’s enduring impact on Brazil has had significant implications for health and oncology outcomes. This historical essay delves into the profound changes brought about by the transatlantic slave trade from Africa to the Americas, particularly in terms of its influence on the economy, sociocultural habits, and health outcomes. This essay explores the enduring connections between the colonial period’s operational dynamics in Brazil and the current epidemiological panorama of head and neck cancer (HNC). The examination provides original insights on the role of tobacco and alcohol production and consumption, alongside the investigation of structural racism, which contributes to disparities in access to diagnosis, treatment, and prognosis for patients with HNC. This article presents novel visions and an analysis of evidence-based strategies to disrupt the adverse impact of colonialism’s legacy on the epidemiology of HNC in Brazil.

## Introduction

Eduardo Galeano, in his work “The Open Veins of Latin America: Five Centuries of the Pillage of a Continent”, asserts, “*History is a prophet who looks back: because of what was, and against what was, it announces what will be*.”^1^ This statement serves as an encapsulation of the book’s essence, condemning the exploitation of Latin America and the Caribbean (LAC). Galeano’s message remains pertinent in the contemporary context, highlighting the relevance of his critique of chronic exploitation in the region. Importantly, Latin American literature reflects this persistent perspective, as exemplified by the concept of the “meaning of colonisation”, which posits that the economic and social organisation of the colony engendered enduring atavisms in modern Brazilian society.^2^ More recently, drawing inspiration from the new institutional economics, Acemoglu and Robinson (2012)^3^ contributed significantly to this discourse, reinforcing the idea of path dependence by comparing the trajectories of the United States and Latin American countries.

LAC comprises 33 diverse nations, including Brazil, Argentina, and Cuba, each undergoing distinct historical processes of formation, independence, economic development, and cultural identity evolution.^4^ Despite differences, a common thread is the lasting socioeconomic inequality, particularly present in the tropical production areas. Over centuries, European colonisers extensively explored and influenced these nations, shaping their trajectories.^1^^,^^5^^,^^6^ Colonialism in LAC, the period from 1494 to 1824 years CE,^1^ significantly impacted the economy, social values, and cultural habits of these countries, influencing the epidemiology of diseases such as head and neck cancer (HNC),^1^^,^^6^ a heterogeneous group of malignant neoplasms that affect the oral cavity, oropharynx, hypopharynx, larynx, nasopharynx, and salivary glands, among other anatomical locations.^7^^,^^8^ The main etiological factors are chronic tobacco and alcohol consumption for oral cavity tumours, sun exposure for lip cancer, and infections by oncogenic viruses such as Epstein-Barr Virus and Human Papillomavirus.^7^^,^^8^ GLOBOCAN 2020 reports Brazil, Argentina, and Cuba with the highest HNC prevalence in LAC,^9^ reinforcing that this epidemiological scenario is not a mere coincidence but a result of historical legacies influencing contemporary health outcomes.

The colonial era in Brazil (1500–1822 years CE)^1^ influenced the economic landscape and public health. The arrival of European exploiters and the influx of enslaved individuals from Africa reshaped the evolution and epidemiology of communicable and non-communicable diseases.^10^ Tuberculosis, caused by *Mycobacterium tuberculosis* (MTB), is an interesting example, since there’s evidence indicating that MTB arrived in Latin America with the European explorers.^10^ Conversely, the profit crops of tobacco and sugarcane imposed during the colonial period shaped the trends of non-communicable diseases, including HNC. This influence, whether direct or indirect, is noteworthy, especially considering that Brazil and Cuba exhibit the highest rates of HNC in LAC^8^^,^^11^ Both countries were leading producers of tobacco and sugarcane for distilled beverages during the colonial era.^11^^,^^12^ The culture of tobacco and alcohol consumption persists in these countries impacting HNC epidemiology. Notably, 65% of HNCs in Brazil, Argentina, and Cuba were attributed to alcohol and tobacco consumption, underscoring the critical role of addressing these behavioural risk factors in the region.^13^

While research has focused on the biological facets of HNC, crucial for refining therapeutic approaches, a gap exists in exploring sociohistorical determinants influencing epidemiological patterns and the challenges impeding effective HNC prevention. The exploration of health dynamics in the context of colonisation finds further depth in the work of Acemoglu et al. (2001),^3^ which delves into the intricate relationship between health outcomes and historical colonisation by leveraging variations in European mortality rates. Notably, European colonisation policies varied significantly across different colonies, giving rise to distinct institutions. By utilizing disparities in European mortality rates as a variable, the study reveals insights into the ongoing discourse on the complex interplay between historical processes, institutional frameworks, and their lasting impact on health disparities.^3^

The current scientific landscape is characterised by a lack of studies delving into the multifaceted sociohistorical dimensions in LAC countries. This scarcity presents an opportunity to foster a more comprehensive understanding of the contextual factors influencing the challenging prevention paradigm of HNC in the region.^5^^,^^7^, ^8^, ^9^ Despite the complexity of measuring the impact of colonialism on health and cancer epidemiology, owing to a lack of well-documented historical evidence, this review contends that colonialism has indeed left its mark on the epidemiology of HNC in Brazil. Furthermore, the interconnected history of HNC in other LAC countries, such as Cuba and Argentina, is explored, warranting their inclusion in the discussion.

Of importance is the examination of the role played by the influential tobacco and sugarcane industries, as well as the enduring legacy of the transatlantic slave trade in Brazil. This analysis extends to ongoing public policies designed to disrupt this historical legacy, proposing future directions to enhance these policies, and fostering a more nuanced understanding of the complex interplay between historical factors and the contemporary landscape of HNC in Brazil and other parts of the LAC region.

**Search strategy and selection criteria**

To gather references for this review, searches were conducted on Medline/PubMed (US National Library of Medicine, Bethesda, USA), SCOPUS (Elsevier, Amsterdam, The Netherlands), EMBASE (Elsevier, Amsterdam, The Netherlands), and LILACS (*Literatura Latino-Americana e do Caribe em Ciências da Saúde*) using the search terms ‘squamous cell carcinoma of the head and neck’, ‘Latin America’, ‘Brazil’, ‘tobacco’, ‘alcohol’, ‘colonialism’, and ‘slavery’, without any date restrictions. These terms were adapted for each database. Additionally, we searched the authors’ files, reference lists, and relevant books. We included papers published in English, Portuguese, and Spanish. Two authors (BNFLM and ESS) independently reviewed the titles and abstracts of all references identified in the electronic databases and selected the articles that met the inclusion criteria. The present study is a personal view based on the originality and relevance of the final reference list constructed by the authors.

## The past

### Impact of tobacco in the colonial period

The historical narrative of tobacco in LAC is intricate and spans several millennia. Originating as an herb native to American civilizations, including the Incas and the Tupi Indigenous Community, the earliest recorded use of tobacco dates back to 6000–8000 years BCE, particularly in the territories corresponding to present-day Brazil.^14^^,^^15^ The Tupi Indigenous Community utilized tobacco predominantly in religious rituals and therapeutic practices, employing its properties to manage pain, head and neck infections, dental issues, and various other conditions.^14^^,^^15^ With the arrival of Portuguese and Spanish colonisers in the Americas, the Tupi Indigenous Community offered tobacco as a valuable commodity during negotiations.^14^ Historical accounts point to Christopher Columbus as one of the earliest observers of Indigenous tobacco consumption, recognizing its cultural significance in the region.^14^ The introduction of tobacco to Europe occurred in 1542 through Portuguese and Spanish efforts, but it took approximately a century for the herb to be fully integrated into European culture.^14^^,^^15^ The popularity of tobacco surged when Queen Catherine de Medici of France, seeking relief from migraines, adopted the herb on the recommendation of diplomat Jean Nicot. The genus to which the tobacco plant belongs, *Nicotiana*, is named after Nicot, giving rise to the term “nicotine”.^14^ Subsequently, tobacco became a widespread habit among royalty and disseminated across Europe and the globe, becoming intertwined with notions of health, emancipation, and sophistication.^14^

Tobacco seeds were introduced around the world from the 16th century onwards.^12^^,^^14^ However, production of the herb in the Americas remained preferred, resulting in a marked increase in tobacco production on the continent and a valuable commodity worldwide.^12^ In terms of export values, tobacco was the fourth most exported product by Brazil in the final decades of the 18th century, and it was extensively used in the slave trade with Africa.^12^ The economic power of the tobacco industry has left emblematical marks on the construction of Brazil, for example, the flag of the Iberian Union period in Brazil (1616–1640),^16^ the imperial flag of Brazil (1822–1889)^17^–the first flag of independent Brazil–and the current coat of arms of Brazil, since November 15, 1889, all feature the representation of the tobacco branch.^18^

Hence, it is crucial to acknowledge the complexity in addressing primary prevention of HNC through tobacco control and regulation in Brazil, the historical birthplace of tobacco consumption and the epicentre of tobacco production. Brazil has commendably implemented public policies to combat tobacco consumption, resulting in lower smoking rates in comparison to the United States and Europe,^19^ however, among high-risk populations for HNC, including men with lower income and African Brazilians, smoking rates remain disproportionately high.^20^^,^^21^ This correlation is not coincidental, as it mirrors the elevated incidence and mortality rates of HNC in Brazil.^7^^,^^20^^,^^21^

### The culture of sugarcane and *cachaça*: alcohol as a powerful weapon of colonisation

The historical trajectory of alcohol consumption in human civilization is profound and complex. Alcoholic beverages have been integral to various societal contexts, including religious rituals, festivities, and agricultural labour activities, since the inception of recorded history.^22^, ^23^, ^24^ Amidst the complexities and historical nuances, alcohol played a pivotal economic role during the era of European colonial expansion.^22^, ^23^, ^24^ It emerged as a potent instrument of colonialism, wielded strategically due to its potential to induce addiction and, consequently, foster dependence on colonizers for the procurement of additional products.^22^

In the historical context of Brazil, the cultivation of sugarcane held greater economic significance than tobacco production. Alcohol, a key product derived from sugarcane, played a pivotal role in the triangular trade connecting Europe, Africa, and the Americas during the era of colonial expansion. This trade network involved the transportation of enslaved individuals to the Americas to toil on sugarcane plantations, providing the raw material for the production of rum in Europe.^12^ Brazil emerged as one of the foremost producers and exporters of alcoholic beverages, with a particular emphasis on *cachaça*, from the 16th to the 18th centuries.^25^, ^26^, ^27^, ^28^, ^29^, ^30^ While *cachaça* production was widespread across Brazil, sugar mills located in the states of Rio de Janeiro and Minas Gerais stood out as major contributors to the production of *cachaça* for both trade with Africa and the domestic market.^31^^,^^32^ Notably, by the year 1695, Brazil had attained the status of the world’s largest spirits distiller.^25^, ^26^, ^27^, ^28^, ^29^, ^30^

The consumption of alcohol by enslaved individuals in Portuguese America exhibited a paradoxical impact. The inhumane conditions of labour, characterised by exposure to intense sunlight, and limited access to food, prompted enslaved workers to consume substantial amounts of *cachaça* as a coping mechanism.^33^ Although the ingestion of alcohol was not conceptualised as a nutritional intervention, it inadvertently provided nutritional and fluidic support to labourers due to the carbohydrate content present in *cachaça*.^33^, ^34^, ^35^ Despite its inadvertent nutritional contribution, *cachaça* was not without complications. Its consumption was associated with addiction, and concerns arose regarding social disorder, rebellious sentiments, ideas of emancipation, and the potential for diseases.^33^ Some authors went so far as to characterise the taste of distilled drinks, including *cachaça*, as a veritable venom for Africans and Native Americans, attributing its consumption to the perpetuation of addiction across time and generations.^22^ Nevertheless, the pervasive influence of alcohol has penetrated the roots of contemporary society, contributing to significant public health challenges.

### The world in movement: transatlantic trade of enslaved people and slave labour

The transatlantic slave trade, driven by economic, social, and political motives, was the most significant forced displacement in history. Portugal and Spain were the first to initiate the trade, motivated to establish a lucrative sugarcane industry in LAC.^36^ Other countries, such as France and the United Kingdom, were also involved in the transatlantic slave trade.^36^ It is estimated that more than 11 million Africans were forcibly displaced to the LAC to labour in the cultivation of tobacco, sugarcane, coffee, rice, cocoa, and cotton and in the exploration of silver and gold.^36^ Over 2 million enslaved Africans died during the more than 36,000 transatlantic voyages to trafficking them.^36^ Brazil had the most significant number of enslaved people in history, having received over 5 million (47%) African captives.^36^ The cultural practices promoted during this period, primarily driven by economic motives, have impacted the incidence and prevalence of many diseases in the contemporary world.^6^

The transatlantic slave trade had a profound impact on the Caribbean Islands and Brazil, both emerging as focal points of its consequences.^36^ Enslaved men, women, and children in these regions bore the weight of forced labour, primarily on sugarcane plantations that held economic dominance. The Caribbean Islands, additionally, played a notable role as major producers of tobacco during this period. Cuban tobacco garnered global recognition for its quality characterized by an intense taste, aroma, and combustibility.^12^^,^^37^

The establishment of slave monocultures in LAC during the transition to the 19th century, with a particular focus on tobacco and sugar, despite the introduction of new commodities such as cotton and coffee, served to solidify the unequal economic and social landscapes of Brazil and Cuba.^38^ This failure to instigate social transformations resulted in persistent inequality and racism that transcended generations, presenting a tough challenge to societal integration. Therefore, a substantial segment of the population, primarily composed of Black and economically disadvantaged individuals, remained distanced from essential health services.^39^

## The present

### The influence of the colonial period on HNC in Brazil

The colonial era exerted a profound influence on the societal values, economy, cultural practices, religion, and health of Brazil and other nations in LAC, instigating intricate transformations in disease patterns as a consequence of interactions among Native Americans, Europeans, and Africans.^39^ Notably, the discovery, production, exportation, consumption, and popularisation of tobacco and distilled alcohol played a pivotal role in the heightened prevalence of HNC in Brazil, given that alcohol and tobacco stand as predominant risk factors.^7^^,^^9^ This increased prevalence is tied to the enduring colonial legacy of distilled alcohol and tobacco consumption, ingrained in the economic fabric of these societies.^40^ It is crucial to underscore that a nation’s role as a producer does not necessarily align with high levels of domestic consumption. Nevertheless, the impact of production on a country’s health outcomes is substantial, particularly when such products hold historical significance in cultural and economic contexts. To illustrate, China stands as the foremost global producer of tobacco, boasting one of the highest prevalence rates of smoking (23% of the population), and ranks third globally in terms of oral cancer prevalence.^41^

Patterns of tobacco and alcohol consumption share commonalities among nations historically implicated in the transatlantic slave trade.^42^^,^^43^ Remarkably, in the year 2020, the prevalence of tobacco use among adults displayed consistency across Brazil, Cuba, France, Spain, and the United Kingdom.^43^ Similarly, in 2016, the incidence of alcohol consumption exhibited a comparable trend among these nations.^42^ The transatlantic voyages exerted significant health ramifications on both sides of the Atlantic, impacting Americans, Africans, and Europeans similarly. European nations engaged in the slave trade, including Portugal, Spain, France, and the United Kingdom, exhibit elevated crude incidence rates of lip and oral cancer. The overall crude incidence rate for Europe stands at 8.7, whereas for the aforementioned countries, it surpasses 10.6.^9^ While cancer incidence is a complex interplay of genetic, epigenetic, and environmental factors, precluding the attribution of the heightened cancer incidence solely to a single historical event, it is plausible to assert that transatlantic voyages played a contributory role by facilitating alcohol and tobacco consumption, predisposing Native Americans and Europeans to HNC.^44^, ^45^, ^46^

The concept of racial domination, inherently Eurocentric, stands as a distinctive trait of colonialism.^47^ The term ‘race’ was seldom employed prior to the Navigation Acts, emerging as a deliberate human construct designed to propagate the unfounded belief in the intrinsic superiority of white individuals over their non-white counterparts.^47^ Motivated by this ideology of racial supremacy, colonisers perceived the colonised as inherently inferior.^47^ This notion has endured through centuries, imprinting societal values that underpin one of colonialism’s most reprehensible legacies: structural racism. This enduring historical stain persists into contemporary times, evidenced by elevated levels of poverty, social and political exclusion, access barriers, and compromised health outcomes experienced by non-white populations.^47^

Structural racism refers to formalising a set of institutions, culture, history, and ideology that generate and maintain inequity among ethnic groups, contributing to disparate health outcomes.^48^ Scientific elites and governments in LAC have promoted structural racism by encouraging the ‘whitening racial strategy’.^49^ This strategy was used in many countries, such as Argentina and Brazil, to motivate the immigration of Europeans to promote an admixed population driven by eugenic idealism.^49^ In Brazil, the main objective of this campaign was to bring European labourers for agricultural activity after the abolition of slavery. The scientific elites supported the idea of the racial superiority of Europeans, preferring whites to free Afro-descendants on coffee plantations.^49^^,^^50^

Interestingly, the above-mentioned Europeans that came to Brazil and worked hard in the labour of coffee were exposed to intense sunlight, influencing the epidemiology of lip cancer in the country. For example, studies have shown a high incidence of lip cancer in the southern states of Brazil where there’s a high number of European descendants among rural workers,^51^ on the other hand, in the northeast states (with a high number of African descendants among rural workers), the tongue is the most frequent oral cancer site, consistent with the global panorama^52^ These epidemiological differences in oral cancer between the south and north reinforce that the colonial period has impacted the trends of HNC in Brazil.

### The role of the Brazilian health system

The trajectory of Brazil’s national health history underwent a profound transformation in the late 1980s with the establishment of the Unified Health System (SUS), providing healthcare services for all Brazilian citizens. The implementation of SUS constitutes a cooperative endeavour involving the Federal Government, individual states, and municipalities, reflecting a concerted commitment to public health.^53^ With a broad scope of services, SUS extends its coverage across primary care, intermediate and high-complexity healthcare, emergency services, hospital care, epidemiological oversight, sanitary measures, environmental surveillance, and pharmaceutical assistance.^53^ Positioned as one of the world’s largest public health systems, SUS stands as a flagship for comprehensive healthcare delivery, ensuring universal and cost-free accessibility for the entire population while emphasizing the provision of holistic health services. Despite the availability of private healthcare options in Brazil, a noteworthy majority, approximately 75% of patients, exclusively rely on SUS for their healthcare needs.^54^

In this context, extended wait times for health services, create a challenging landscape in which timely healthcare access becomes more than a logistical inconvenience–they emerge as critical factors contributing to the delayed diagnosis of HNC, which, in turn, sets the stage for disease progression and a less favourable prognosis. HNC patients seeking care within the SUS face a prolonged journey from symptom onset to treatment initiation, as highlighted by a recent study.^55^ The mean time from the onset of symptoms to the commencement of treatment was 217 days. Notably, the most extended interval was attributed to professional delay, primarily arising from initial consultations with general practitioners and misdiagnosis. This research highlights critical areas for improvement in the healthcare system to enhance timely access to diagnosis and treatment for HNC patients,^55^ including possible differences in the diagnostic and therapeutic itinerary for African Brazilian populations, as previously observed among other African American communities.^56^ In the Brazilian context, where the ‘60-day law’ facilitates that individuals diagnosed with malignant neoplasms receive their initial treatment within 60 days within the public healthcare system, the impact of timely healthcare access gains heightened significance.^57^

The treatment for HNC involve surgery, radiotherapy, and chemotherapy.^58^ In Brazil, the lack of access to radiotherapy within the SUS represents a severe challenge, affecting approximately 73,000 cancer patients annually and totalling 1.1 million individuals from 2008 to 2022.^59^ This deficit directly contributed to over 110,000 deaths, as per the Brazilian Society of Radiotherapy report.^59^ In a scenario where radiotherapy is crucial for about 60% of cancer patients, the scarcity of this resource negatively impacts both patients, depriving them of proper treatment and allowing the disease to progress, and the healthcare system, increasing costs to treat advanced stages of cancer.^59^ The insufficient number of radiotherapy units, many of which are obsolete, coupled with the outdated payments from SUS for treatments, result in limited access and less effective therapies.^59^

Brazil hosts the largest population of African descent outside the African continent, comprising over 100 million individuals identified as Brown or Black, constituting more than 50% of the total population.^39^ Despite the nation’s diverse demography, structural racism is entrenched within the core of the Brazilian health system. This systemic issue contributes significantly to healthcare disparities, particularly within the domain of cancer-related aspects such as early diagnosis, treatment, and survival rates. Recent data underscores that, in comparison to other demographic groups, Black Brazilians encounter heightened exposure to both smoking and alcohol consumption.^21^ This aligns with the observation that individuals identifying as Black Brazilians receive diagnoses at the most advanced HNC stages,^60^ displaying elevated rates of disease progression and mortality related to oral and oropharyngeal cancer.^60^ In a parallel clinical context, African Brazilian men confront a 300% higher risk of prostate cancer metastasis at the time of diagnosis when compared to their white counterparts.^61^

Decolonisation, as a global movement, advocates for dismantling the enduring legacies of colonialism within the realms of science and health, with a primary focus on fostering equity and justice.^62^ A pivotal facet of this endeavour involves eradicating racism entrenched within medical education. Notably, disparities in the treatment of patients based on skin colour persist, exemplified by instances where physicians handle individuals with identical symptoms differently depending on their ethnical background.^63^ In the domain of oncology, racial inequities are evident, as evidenced by studies indicating that white women are more likely to receive information regarding the impact of family history on breast cancer compared to their non-white counterparts.^64^ This trend extends to late-stage diagnoses, particularly exemplified in Brazil, where an analysis revealed that Black women face a 20% higher likelihood of presenting an advanced clinical stage of cervical cancer compared to other ethnic groups.^65^ The impact of racial disparities continues into palliative care, where differences in access based on racial and ethnic backgrounds persist.^66^ Historical prejudices, such as the false notion propagated by enslavers during the colonial period that Blacks were less sensitive to pain, persist in influencing contemporary healthcare practices.^67^ Despite lacking scientific merit, these myths perpetuate within health institutions, resulting in enduring racial inequalities in pain management throughout the end-of-life care continuum.^66^, ^67^, ^68^ Recent years have seen some strides in acknowledging and addressing racial biases in medical care. For instance, a recent article has publicly recognized its historical role in perpetuating racial and ethnic bias, reflecting a broader effort to rectify past shortcomings in this critical domain.^69^

## The future

### Looking in the rearview mirror and moving forward: lessons and opportunities

Galeano’s phrase mentioned at the beginning of this manuscript invites us to reflect on the past, learn from it, move towards understanding, and uproot colonial practices in healthcare.^1^ Developing strategies for decolonisation is a complex process that involves cultural, economic, political, social, religious, and specific aspects in each country. However, some reflections are important. Therefore, a set of scientific-based directions that are contributing to the mitigation of the historical impact of colonialism on oncology, including trends of HNC in Brazil, is presented.

#### Public policies for tobacco control measures

The first movement towards tobacco control in Brazil began in the 1960s.^70^ In this context, an essential step in the 21st century was The World Health Organization (WHO) Framework Convention on Tobacco Control, which provides strategies for countries to manage and implement tobacco control measures. In this context, the WHO introduced the MPOWER measures, which are applied worldwide and consist of monitoring tobacco use.^71^ MPOWER is an acronym for the following measures: M—Monitor tobacco use and prevention policies; P—Protect people from tobacco smoke; O—Offer help to quit tobacco use; W—Warn about the dangers of tobacco; E—Enforce bans on tobacco advertising, promotion and sponsorship; R—Raise taxes on tobacco. Despite the challenges, Brazil has been successful in implementing all the six measures of the MPOWER as indicated in a recent WHO report.^71^ It is vital to enhance these existing efforts among high-risk populations for HNC, including men with lower income and African Brazilians where smoking rates remain high.^20^^,^^21^

#### Enforce public policies that discourage chronic alcohol use

In Brazil, the year 2021 witnessed the recording of more than 400 thousand appointments by the SUS for individuals grappling with mental and behavioural disorders attributable to drug and alcohol use, marking a 12% surge in comparison to the previous year. SUS is committed to providing comprehensive care and ongoing support for individuals affected by any form of substance dependence, with Primary Health Care assuming a pivotal role in the management of such cases. Augmenting the healthcare framework are specialized centres, including the Psychosocial Care Centre, designed specifically for addressing issues related to substance abuse.^72^ Notwithstanding these efforts, recent data from 2018 indicates that the average alcohol consumption among Brazilian adults reached 7.4 L per capita, exceeding the LAC average of 6.9 L.^73^ Hence, although Brazil has implemented significant and efficacious alcohol control policies, such as the drink-driving policy, there remains room for enhancement.^73^ It is vital to broaden the scope of public policies addressing alcohol control, with a specific focus on populations at elevated risk for HNC.

#### Enhance representation of black individuals in healthcare institutions

A recent study demonstrated a 50% reduction in the mortality of Black newborns when attended to by Black physicians, suggesting a tangible benefit of physician-patient self-identification in mitigating racial disparities within hospital settings.^74^ Responding to the imperative to address health inequities affecting the Black population, the National Policy for Comprehensive Health of the Black Population has been established by the Brazilian Ministry of Health. This policy acknowledges that the living conditions of this population stem from unjust historical social, cultural, and economic processes.^75^ Integrated into the dynamics of the SUS, it employs collaborative and participatory management strategies. These encompass the utilisation of racial classification in epidemiological information production for priority setting and decision-making, the augmentation and fortification of social control mechanisms, and the formulation of actions and strategies to identify, rectify, combat, and prevent institutional racism within workplaces, professional training, and ongoing education processes. The implementation of additional affirmative actions of a similar nature will help to prioritise and achieve health equity and promote racial equality for HNC diagnosis and treatment in Brazil.^75^

## Limitations

This study is subject to limitations that warrant acknowledgment. The availability of historical data to establish more precise associations is limited. Most investigations into ‘racial disparities’ in oncology are predominantly conducted within North America, particularly the United States. Consequently, extrapolating these correlations to Brazil and other countries in the LAC region poses a challenge. Within the internal landscape of LAC, variations in colonisation processes and routes further complicate the generalizability of findings. Nevertheless, it is crucial to recognize the role played by commercial assets, notably tobacco, sugarcane, and slavery, in the colonisation and exploration dynamics within this region. Despite these inherent limitations, this study provides valuable insights into the discernible influence of colonial heritage on HNC trends in Brazil.

## Conclusions

The repercussions of colonialism have exerted profound influences on various facets of Brazil, encompassing its societal values, economic structure, cultural fabric, and public health landscape. The historical cultivation of sugarcane and tobacco crops, coupled with the transatlantic trade of enslaved individuals, manifests a nuanced connection with the epidemiology of HNC in the region, persisting to the contemporary era. The enduring heritage of the tobacco and sugarcane industries, which emerged during the colonial period, has posed persistent challenges in decreasing HNC incidence. Furthermore, the entrenched promotion of ‘racial supremacy’ by colonizers has become deeply embedded in Brazil, fostering structural racism that detrimentally affects equitable healthcare access, contributes to disparaging trends in HNC epidemiology, and impacts survival rates. It is imperative to dismantle the entrenched roots of colonialism in Brazil to foster parity in healthcare access, particularly in the realm of head and neck oncology.

## Contributors

Beatriz Nascimento Figueiredo Lebre Martins: Conceptualization, investigation, methodology, writing- original draft.

Erison Santana Dos Santos: Conceptualization, investigation, methodology, writing- original draft.

Felipe Paiva Fonseca: Data curation, validation Writing- Reviewing and Editing.

William Nassib William Jr: Validation, Writing- Reviewing and Editing.

Thiago Bueno de Oliveira: Writing- Reviewing and Editing.

Gustavo Nader Marta: Writing- Reviewing and Editing.

Aline Lauda Freitas Chaves: Visualization, Investigation, Writing- Reviewing and Editing.

Ana Carolina Prado Ribeiro: Writing- Reviewing and Editing.

Lekan Ayo-Yusuf: Data curation, Writing- Reviewing and Editing.

Maria Paula Curado: Investigation, Writing- Reviewing and Editing.

Alexandre Macchione Saes: Validation, Writing- Reviewing and editing.

Luiz Paulo Kowalski: Writing- Reviewing and Editing.

Alan Roger Santos-Silva: Conceptualization, data curation, formal analysis, investigation, methodology, project administration, supervisor, validation, Writing- Reviewing and Editing.

## The use of AI and AI-assisted technologies statement

AI and AI-assisted technologies, specifically ChatGPT/OpenAI, were employed in the refinement of this manuscript to enhance clarity and streamline the textual structure. Following the utilization of these tools, the authors meticulously reviewed and edited the content, assuming full responsibility for the final publication. OpenAI’s GPT-3.5 language model was used on December 28, 2023.

## Declaration of interests

We, the authors of this manuscript, declare that there is no financial relationship with any commercial association, current or within the past 2 years, which might pose a potential, perceived, or real conflict of interest. This includes grants, patent-licensing arrangements, consultancies, stock or other equity ownership, advisory board memberships, or payments for conducting or publicizing our study. The authors also state the material is original, has not been published elsewhere, and is being submitted only to **The Lancet Regional Health—Americas**.

## Appendix A Supplementary data

Translated Abstract_EP

List of names of the study group members
