# Supplementary material for: The impact of colonialism on head and neck cancer in Brazil: a historical essay focussing on tobacco, alcohol and slavery
Source: Lancet Reg Health Am. 2024 Feb 9;31:100690. doi: 10.1016/j.lana.2024.100690 (PMC10873724; doi:10.1016/j.lana.2024.100690)
Supplement: Translated Abstract_EP [file mmc1.docx]

*Editor note: These translations in Portuguese and Spanish were submitted by the authors and we reproduce it as supplied. It has not been peer reviewed. Our editorial processes have only been applied to the original abstract in English, which should serve as reference for this manuscript.*

**RESUMO**

O impacto duradouro do colonialismo no Brasil teve implicações significativas na saúde e nos resultados da oncologia. Este ensaio histórico investiga as profundas mudanças provocadas pelo comércio transatlântico de escravizados da África para as Américas, particularmente em termos de sua influência na economia, nos hábitos socioculturais e nos resultados de saúde. Este ensaio explora as conexões duradouras entre a dinâmica operacional do período colonial no Brasil e o atual panorama epidemiológico do câncer de cabeça e pescoço (CCP). O exame fornece percepções originais sobre o papel da produção e do consumo de tabaco e álcool, juntamente com a investigação do racismo estrutural, que contribui para as disparidades no acesso ao diagnóstico, ao tratamento e ao prognóstico dos pacientes com CCP. Este artigo apresenta novas visões e uma análise de estratégias baseadas em evidências para interromper o impacto adverso do legado do colonialismo na epidemiologia do CCP no Brasil.

**Palavras-chave:** Câncer; Neoplasias de cabeça e pescoço; Epidemiologia; Brasil; Etnia; Prognóstico; Mortalidade; Tabaco; Álcool; Colonialismo.

**RESUMEN**

El impacto perdurable del colonialismo en Brasil ha tenido importantes repercusiones en los resultados de salud y oncológicos. Este ensayo histórico se adentra en los profundos cambios provocados por la trata transatlántica de esclavos de África a las Américas, especialmente en lo que respecta a su influencia en la economía, los hábitos socioculturales y los resultados sanitarios. Este ensayo explora las conexiones perdurables entre la dinámica operativa del periodo colonial en Brasil y el panorama epidemiológico actual del cáncer de cabeza y cuello (CCC). El ensayo ofrece perspectivas originales sobre el papel de la producción y el consumo de tabaco y alcohol, junto con la investigación del racismo estructural, que contribuye a las disparidades en el acceso al diagnóstico, el tratamiento y el pronóstico de los pacientes con CCC. Este artículo presenta visiones novedosas y un análisis de estrategias basadas en la evidencia para interrumpir el impacto adverso del legado del colonialismo en la epidemiología del CCC en Brasil.

**Palabras-clave:** Cáncer; Neoplasias de cabeza y cuello; Epidemiología; Brasil; Etnia; Pronóstico; Mortalidad; Tabaco; Alcohol; Colonialismo.
