# Supplementary material for: The impact of colonialism on head and neck cancer in Brazil: a historical essay focussing on tobacco, alcohol and slavery
Source: Lancet Reg Health Am. 2024 Feb 9;31:100690. doi: 10.1016/j.lana.2024.100690 (PMC10873724; doi:10.1016/j.lana.2024.100690)
Supplement: List of names of the study group members [file mmc2.docx]

**List of names of the study group members**

**Latin American Cooperative Oncology Group, Brazilian Group of Head and Neck Cancer**

| William Nassib | William Jr. |
| --- | --- |
| Thiago Bueno | de Oliveira |
| Gustavo Nader | Marta |
| Aline Lauda | Freitas Chaves |
| Maria Paula | Curado |
| Luiz Paulo | Kowalski |
| Alan Roger | Santos-Silva |

William Nassib William Jr MD, MSc, PhD^c,d^

Thiago Bueno de Oliveira MD, MSc, PhD^e^

Gustavo Nader Marta MD, MSc, PhD^f^

Aline Lauda Freitas Chaves MD, MSc^d,g^

Maria Paula Curado MD, PhD^k^

Luiz Paulo Kowalski MD, PhD^m,n^

Alan Roger Santos-Silva, DDS, MSc, PhD^a^

^a^University of Campinas (UNICAMP), Oral Diagnosis Department, Piracicaba Dental School, Piracicaba, São Paulo, Brazil.

^c^Centro Oncológico BP, Beneficência Portuguesa de São Paulo, Brazil.

^d^Latin American Cooperative Oncology Group, Brazilian Group of Head and Neck Cancer, Brazil.

^e^Medical Oncology Department, AC Camargo Cancer Center, São Paulo, SP, Brazil.

^f^Department of Radiation Oncology, Hospital Sírio-Libanês São Paulo, São Paulo, Brazil.

^g^DOM Oncology Group, Divinópolis, Minas Gerais, Brazil.

^k^Epidemiology AC Camargo Cancer Center, São Paulo, SP, Brazil.

^m^Department of Head and Neck Surgery and Otolaryngology, A C Camargo Cancer Center, São Paulo, Brazil.

^n^Department of Head and Neck Surgery, University of São Paulo, Brazil.
